# Inter-rater reliability of vehicle color perception for forensic intelligence

**DOI:** 10.1371/journal.pone.0218428

**Published:** 2019-06-18

**Authors:** Khai Lee, Anis Amanina Abdul Fatah, Nuryuhanis Mohd Norizan, Zakiah Jefrey, Fatin Hanani Md Nawi, Wan Fatihah Khairunisa Wan Nor, Huan Xin Wong, Saiful Fazamil Mohd Ali, Poh Ying Lim, Kah Haw Chang, Ahmad Fahmi Lim Abdullah

**Affiliations:** 1 Forensic Science Program, School of Health Sciences, Health Campus, Universiti Sains Malaysia, Kubang Kerian, Kelantan, Malaysia; 2 Faculty of Resource Science and Technology, Universiti Malaysia Sarawak, Kota Samarahan, Sarawak, Malaysia; 3 School of Marine and Environmental Sciences, Universiti Malaysia Terengganu, Kuala Terengganu, Terengganu, Malaysia; 4 Department of Chemistry, Faculty of Science, University of Malaya, Kuala Lumpur, Malaysia; 5 Criminalistic Section, Forensic Division, Department of Chemistry, Petaling Jaya, Selangor, Malaysia; 6 Department of Community Health, Faculty of Medicine and Health Sciences, Universiti Putra Malaysia, Serdang, Selangor, Malaysia; Fordham University, UNITED STATES

## Abstract

The topcoat color of motor vehicles offers vital information while investigating vehicular accidents, especially in instance of hit-and-run, since witnesses seldom perceive and retain the plate details. Differences in color perceptions among individuals with normal vision may lead to confusion in determining the color of the car involved. In this way, witnesses of crash accidents could potentially initiate flawed leads in forensic investigation, and thus affect the administration of justice. In this study, the inter-rater reliability of vehicle color determination by different volunteers was explored. Six individuals observed the topcoat colors of 500 stationary and 500 moving vehicles from five locations, employing a common system of color gradation. The outcome was binary: the vehicle color was either a “match” or “non-match”. This was followed by statistical analysis in terms of the colors’ frequencies and inter-rater reliability, based on which more suitable color descriptions were determined for subsequent comparisons of stationary and moving vehicles. Higher match frequencies and greater inter-rater reliability were observed when color gradations were disregarded. The frequency of correct matches could have been closely related to their relative on-the-road distribution, regardless of the statuses of observed vehicles. It was also found that black and white were associated with a greater number of matches than were intermediate colors, which should be carefully interpreted during forensic investigation to avoid wrong leads. In conclusion, the present study demonstrated the forensic significance of vehicle topcoat color determination, particularly in cases where witness statements are crucial.

## Introduction

Multiple coatings of automotive paints are applied to vehicles for both protective and decorative purposes [1, 2]. These paints are frequently encountered as trace evidence in vehicular accidents, allowing for the association of questioned samples recovered from a scene with control samples of known sources through forensic examination. In certain vehicular accidents such as hit-and-run, the investigative team would look for the plate number and color information alleged by a witness or victim of the accident in order to begin their investigation. Unfortunately, in cases where witnesses either did not notice or cannot remember plate numbers, the colors of vehicles, the descriptions of which are based solely on the perceptions of witnesses, become the main lead. Therefore, the inability to correctly describe the colors of vehicles may lead investigations astray.

During a trial, investigative officers or forensic scientists are often required to explain the evidential value of questioned paint evidence recovered from a vehicular accident, including its color [3, 4]. Surveys and compilations of vehicle color distributions generalized as topcoat color [5] (technically, unlike the solid paint system, the top layer of a metallic paint system is a colorless clear coat that covers the metallic basecoat) have been conducted to assess the probative value of a vehicle’s color in supporting forensic conclusions [3, 4, 6–11]. In addition, color determination through careful interpretation of submitted paint samples can also be correlated with the color momentarily perceived by a witness during an accident, although this is often difficult. Any lack of accuracy in describing the color perceived by a witness, and especially so if there are two or more witnesses, would impact credibility during cross-examination [12]. Though a standardized color coding system for paint has been established in the forensic community [13], it is not readily available to the public, complicating the process of accurate identification of topcoat color. Previous literature has suggested the possibility of variations in color perception by individuals with normal vision [14–16], supporting the likelihood of such differences generating flawed investigative leads or contradictory testimony.

Inter-rater differences in color determination could be related to categorical differences in observers’ knowledge and perceptions [17]. Color perception is an observer’s ability to perceptually differentiate between colors [18], which could be subjectively affected by personal, cultural, and national beliefs, values, prejudices, and other unknown factors [19]. In view of this, the inter-rater reliability of vehicle topcoat color perception is a topic of interest in forensic intelligence, and the aim of this study was to evaluate inter-rater differences in descriptions of vehicle topcoat color in both static and moving conditions. Based on a prevailing system [3], the topcoat colors of stationary and moving vehicles were surveyed and statistically analyzed. A better color description system was highlighted to increase the agreement percentage among observers. The inter-rater reliability of vehicle color determination among observers was evaluated and colors that could potentially lead to incorrect determination were also identified. To the authors’ knowledge, a survey of this type has not been reported thus far. Such information can serve as an initial lead for investigative teams to verify a witness statement, and subsequently assist forensic teams in tracing the vehicle involved in an accident.

## Materials and methods

### Survey

This study was conducted in five locations within the boundary of the Health Campus of Universiti Sains Malaysia, where a total of 500 stationary and 500 moving vehicles were randomly sampled and studied. Topcoat colors of vehicles were observed at noon in clear weather by six students (aged between 20 and 22 years) from various universities undergoing industrial attachment training in the forensic science program. In this study, only passenger-type vehicles, such as cars and vans, were included; heavy-duty vehicles such as trucks and buses were excluded.

Prior to the survey, the authors conducted an introductory session with the six observers using a standard auto color chart available in forensic laboratories as a guide. This was to assure consistency in color determination, noting the choice of colors available and the color naming system. Buckle et al.’s [3] chart consisting of 29 grades of colors was utilized; this information is depicted in Table 1. Those colors that could not be included in any of the listed colors in Table 1 were counted as “miscellaneous,” as done in our report on vehicle surveys [6].

**Table 1:** Color chart.

No.	Color [3]
1	Black
2	Light blue
3	Medium blue
4	Dark blue
5	Green-blue
6	Light brown
7	Medium brown
8	Dark brown
9	Red-brown
10	Red
11	Red-orange
12	Gold-bronze
13	Light gray
14	Medium gray
15	Dark gray
16	Light green
17	Medium green
18	Dark green
19	Yellow-green
20	Light yellow
21	Medium yellow
22	Dark yellow
23	Maroon
24	Orange
25	Purple
26	Pink
27	White
28	Off-white
29	Miscellaneous

In each location, the six observers simultaneously noted the topcoat color of each car at a distance of approximately two meters. For both stationary and moving vehicles, each observer was given three seconds to write down their observation on a sheet of paper provided. No attempt was made to identify whether a vehicle’s paint system was solid or metallic since this is difficult to determine at a glance. As the survey was conducted within the university, the speed of moving vehicles could not have exceeded 60 km/hr.

The observers’ perceptions were tabulated. Subsequently, the extent of agreement between individuals’ observation regarding topcoat color was checked and interpreted. Regarding agreement, the outcome was binary: it was either a “match” or “non-match.” It was a match when all the observers described the same color code, while it was a non-match when even one observer varied in his/her description of the topcoat color.

### Statistical analysis

Statistical analysis was conducted using Stata software version 12 (StataCorp, USA). Data cleaning and descriptive analyses were performed to ensure there were no errors.

### Evaluation of agreement percentage in relation to color description

Using the colors listed above [3], the frequency and percentage of matches and non-matches in observers’ determinations of topcoat colors were calculated. The statistical output using these colors formed the basic data. Subsequently, shade variations in the colors described in Table 1 were clubbed together to correspond to the basic color, with matches and non-matches calculated in this situation as well.

### Comparison of inter-rater reliability of color determination between two different color descriptions

Inter-rater reliability of color determination for both color descriptions was investigated. Kappa test (κ) statistics were used to assess agreement among the six observers. These values were interpreted as poor agreement (0.00–0.20), fair agreement (0.21–0.40), moderate agreement (0.41–0.60), good agreement (0.61–0.80), and very good agreement (>0.80) [20]. In this study, κ statistic values >0.60 were considered indicative of good inter-rater reliability and <0.60 of poor inter-rater reliability. A p-value <0.05 was considered statistically significant. Based on the statistical output, a color description with better inter-rater reliability was determined. The inter-rater reliability of moving vehicles was also verified using the color description determined in the previous section.

### Determination of “non-match” color combinations among observers

The observational data set was further analyzed to determine the colors with a greater possibility of non-matches among the six observers. The frequency and percentage of the matches and non-matches for each color were demonstrated and compared. Colors that could easily be described differently by the six observers were identified.

## Results

In this study, 214 matches were recorded, which was 72 cases less than the non-matches. This finding indicates that all six observers concurred in the descriptions of only 42.8% of topcoat colors when using the color shades described earlier [3]. Then, basic colors alone were used by totaling the light, medium, and dark colors into one group. For instance, “light gray”‘, “medium gray,” and “dark gray” were all clubbed together as one basic color: “gray.” When the variations in the shades were eliminated, there remained 18 colors and the consequent frequencies of matches increased by 153, constituting of a total of 73.4%.

Inter-rater reliability (κ) values for each color scored in the two calculations (one that included the variations in shades and the other that considered the basic colors) were computed (Table 2). By including the variations in shades as in the prevailing color description [3], black, orange, pink, purple, and light gray recorded very good agreement (κ>0.80), followed by maroon, dark green, white, medium blue, red, light brown, light green, light blue, and dark gray with good agreement (0.61<κ<0.80). These colors demonstrated good inter-reliability (κ>0.60). It was also found that the intermediate colors (i.e. green-blue, yellow-green, red-orange, gold-bronze, and red-brown), as well as miscellaneous shades of basic colors, exhibited poor inter-reliability (κ<0.60).

**Table 2:** κ values of each color in two different color descriptions for stationary vehicles.

Color		Inter-reliability (κ) values**	
		Color chart [3]	Basic color descriptions
Black		0.96*	0.96*
Blue	Light	0.65*	0.81*
	Medium	0.72*	
	Dark	0.44	
Green-blue		0.56	0.56
Brown	Light	0.68*	0.65*
	Medium	0.15	
	Dark	0.44	
Red-brown		0.22	0.22
Red		0.68*	0.68*
Red-orange		0.52	0.52
Gold-bronze		0.32	0.32
Gray	Light	0.89*	0.91*
	Medium	0.45	
	Dark	0.63*	
Green	Light	0.67*	0.79*
	Medium	0.12	
	Dark	0.77*	
Yellow-green		0.52	0.52
Yellow	Light	0.31	0.62*
	Medium	0.40	
	Dark	0.23	
Maroon		0.78*	0.78*
Orange		0.92*	0.92*
Purple		0.91*	0.91*
Pink		0.92*	0.92*
White	Pure white	0.73*	0.98*
	Off-white	0.41	
Miscellaneous		0.40	0.40
**Overall**		0.69* Good agreement	0.85* Very good agreement

*Good inter-rater reliability (κ >0.60)

**Reliability test was significant, *p* <0.05

Using basic color descriptions, the number of matches decreased by 12 when vehicles were in motion (Table 3), but the status of vehicles, either stationary or in motion, did not affect the correct determination of vehicle topcoat colors under the observational conditions. Among the six observers, regardless of whether the vehicles were stationary or moving, white, gray and black were the top three matches and were ranked the same. The high match frequency of the color blue among stationary vehicles was found to decrease when observing moving vehicles. Red was the fourth most frequent match for moving cars. Overall, inter-rater reliability for both stationary and moving vehicles demonstrated very good agreement at κ values of 0.85 and 0.84, respectively.

**Table 3:** Percentages of match and non-match frequencies based on color description.

Color	Stationary vehicles *“Match” (n = 367)*		Moving vehicles *“Match” (n = 355)*	
	Frequency (%)		Frequency (%)	
	Match	Non-match	Match	Non-match
Black	56 (81.2)*	13 (18.8)	56 (76.7)*	17 (23.3)
Blue	26 (44.1)*	33 (55.9)	17 (42.5)*	23 (57.5)
Green blue	1 (20.0)	4 (80.0)	0 (0)	4 (100.0)**
Brown	8 (18.6)*	35 (81.4)	12 (22.2)	42 (77.8)
Red-brown	0 (0)	28 (100.0)**	0 (0)	15 (100.0)**
Red	7 (19.4)	29 (80.6)	30 (58.8)*	21 (41.2)
Red-orange	1 (4.2)	23 (95.8)**	1 (5.6)	17 (94.4)**
Gold-bronze	0 (0)	14 (100.0)**	0 (0)	12 (100.0)**
Gray	120 (70.2)*	51 (29.8)	82 (59.0)*	57 (41.0)
Green	7 (28.0)	18 (72.0)	10 (27.8)	26 (72.2)
Yellow-green	0 (0)	7 (100.0)**	0 (0)	5 (100.0)**
Yellow	3 (23.1)	10 (76.9)	4 (28.6)	10 (71.4)
Maroon	4 (28.6)	10 (71.4)	1 (20.0)	4 (80.0)
Orange	2 (66.7)	1 (33.3)	3 (37.5)	5 (62.5)
Purple	7 (58.3)	5 (41.7)	11 (91.7)	1 (8.3)
Pink	3 (60.0)	2 (40.0)	1 (33.3)	2 (66.7)
White	122 (92.4)*	10 (7.6)	121 (92.4)*	10 (7.6)
Miscellaneous	0 (0)	3 (100.0)**	6 (26.1)	19 (73.9)

* top five “match” scores

** top five “non-match” percentages

In this study, a non-match was scored if there was even a single difference among observers. Therefore, cases involving determination of two or more colors by the six observers were separately recorded as non-matches in their respective color categories. White scored the lowest percentage of non-matches, followed by black and gray. On the contrary, there were colors with only non-matches, such as red-brown, gold-bronze, and yellow-green, where no agreement was achieved among observers. It was also noted that the colors that could lead to differences in color determination were similar, regardless of whether the vehicles were stationary or moving.

## Discussion

The majority of non-matches reported in this study were seen as a consequence of the existence of multiple shades of the same basic color. For example, the six observers did not have mutual agreement in determining “white” and “off-white” where 81 cases, accounting for 28.3% were recorded as non-matches. “Dark gray” and “medium gray,” with non-matches recorded in 22 cases (7.7%), and “light gray” and “medium gray,” with non-matches recorded in 21 cases (7.3%), showed similar trends. The higher proportion of matches reported when considering only the basic color description as compared to when shades were included [3] indicates the value of basic colors.

When shade variations in blue, brown, gray, green, yellow, and white were disregarded, the inter-rater reliability increased considerably. White, which was initially coded separately as “white” and “off-white,” became the most reliable color, replacing black. In the basic color description, only the crossover colors (i.e. green-blue, yellow-green, red-orange, gold-bronze, and red-brown) demonstrated poor inter-reliability (κ<0.60), suggesting differences in agreement on such intermediary shades of colors.

Although an introductory session was conducted to calibrate the observers prior to sampling, the variations in color determination could have been due to differences in their ability to discriminate colors as well as personal experiences [19, 21]. A significant increase in the overall inter-reliability from 0.69 to 0.85 was observed when shades were discarded, resulting in very good agreement. Higher reliability in single-color determination was also achieved by disregarding the shade variations in basic colors. This observation was in agreement with Bae et al. [22], who found that colors were easier described by a single term (e.g. gray) rather than including shades (e.g. light gray, medium gray, or dark gray) since the boundaries between these shades differ among individuals. The suggestion of the possibility of eliminating color-specific biases by merging shades into one color ensures better appreciation of the categorical boundaries of basic colors [22]. This was supported by the better scores and reliability obtained when the different shades of a color were combined.

Since all six individuals in this study made their observations in similar conditions, the use of only basic colors, which ensures good inter-rater reliability, is proposed for describing vehicle topcoat colors during forensic investigations, particularly when recording witness statements. In addition, such basic color-based enquiries would limit the use of jargon, thus conforming to the suggestion that an observer feels more comfortable describing a color in a few words rather than having to rely on a spectrum of colors with broad and hardly discriminable shades [23]. However, it is also important to note that the investigative team should gather as much information as possible from a witness as he/she is able to provide.

Higher match frequencies for white, gray, and black could be linked to the on-the-road topcoat color distribution found in an earlier survey [6]. A greater number of vehicles top-coated with these colors could have been observed during the present survey, accounting for approximately 80% of the total matches. However, it has to be emphasized that the on-the-road distribution of topcoat colors could not be linked to the inter-rater reliability in color determination since certain colors such as pink, orange, and purple that have been reported to have very good inter-rater reliability were not among the common topcoat colors of vehicles in the country [6]. The rarity of a color does not appear to influence inter-rater reliability of color perception.

Good inter-rater reliability indicates greater consistency in the estimation of a phenomenon; in this case, matches during color determination. The use of basic colors has been associated with more consistent determination and less confusion as compared to the use of intermediate colors [24]. In this study too, basic colors like white and black had high inter-rater reliability values of 0.98 and 0.96, respectively, during matches. Contrarily, in the case of colors like green-blue, yellow-blue, red-orange, gold-bronze, and red-brown, poor inter-rater reliability values were attributable to a large percentage of non-matches based on the criterion that disagreement by even a single observer was categorized as a non-match (Table 3). The identification of intermediate colors, such as the combination of red/red-brown, red/red-orange, brown/gold-bronze, and red/red-orange/red-brown, was likely to be incorrect. However, it is important to be aware of the possibility of wrong matches even if a particular color demonstrated good agreement regarding inter-rater reliability value. For example, the determination of the color gray was associated with a relatively large percentage of non-matches (29.8%), wherein gray could be confused with blue, brown, or even white.

This study suggests that witness statements regarding intermediate colors should be carefully interpreted during forensic investigations to avoid following wrong leads. Additionally, exact agreement among the observers which did not occur, particularly in determination of intermediate color, could have been due to personal variations [18, 19]. This is exemplified by the non-matches for white (Table 3), which appears unique and the least confusing. While it is highly likely that such a color will be correctly determined by a witness, it is not certain because of differences in individual perception [17, 18, 22]. In fact, the outcome of this study could aid in investigative procedures where a search for a vehicle of a specific color can be broadened to other possibilities whenever a witness can provide more detailed color information.

According to Bae et al. [22], an observer’s visual system can spontaneously assign category labels to signals that interact with encoded shade content to produce bias during response, particularly when an observer is required to describe the vehicle topcoat color after having seen it just once. In other words, observations regarding moving vehicles could lead to delays in encoding colors, which is unlikely to happen for stationary vehicles; the consequently greater bias could be the cause for the slightly lower inter-reliability values among moving vehicles [22]. In this study, although the observational results demonstrated a slight decrease in the overall inter-rater reliability for moving vehicles, a significant association between the matches in color determination was lacking for both stationary and moving vehicles.

Previous literature suggests that memory retrieval delays could affect observers’ color perception [25, 26], especially because of distractors such as surface illumination and the motion of an object [27, 28]. In this study, possible distortions or biases caused by delayed memory were minimized through the provision of sufficient time to assign a color to the moving vehicles, leading to no significant effect on observers’ color perception. Short-term memory could be one factor leading to variations in color determination [28, 29]; nonetheless, future studies on its relationship with color perception, which could offer useful retrievable information to law enforcement authorities, are recommended.

This study was conducted in optimal rating conditions with adequate illumination at a fixed distance for the young observers to determine topcoat colors. However, further collation of information, including vehicle color perception under different conditions, particularly accounting for environmental factors and observers’ vision and attention toward color determination, is necessary for broader forensic intelligence. However, percentage of agreement among colors with greater number of observers observing on the smaller number of objects could be proposed, perhaps in subsequent studies upon the determination of suitable color system and combination of “non-match” colors identified in the current study.

In general, the frequency of matches in topcoat color determination, both for stationary and moving vehicles, could be related to their relative on-the-road distribution. This study indicated that using basic colors without shade variations could lead to better determination of color by an observer, resulting in a greater frequency of matches. The motion of vehicles did not have much effect on the scoring a match, given that the environmental conditions were adequate for an observer to encode the color. This study supports that for forensic intelligence purposes, cases involving descriptions of vehicle topcoat colors shall need greater investigative efforts including a more careful interpretation of witness testimony. It should also be emphasized that individual differences could lead to differences in color perception, especially when involving intermediate colors.

## Conclusion

A survey to investigate the inter-rater reliability of color determination among observers who visually perceived the topcoat colors of both stationary and moving vehicles indicated that the frequencies of matches, and subsequently inter-rater reliability of color determination among observers, significantly increased when using basic color descriptions, disregarding their shades. White and black had the greatest matches, while intermediate colors like green-blue, yellow-green, red-orange, gold-bronze, and red-brown were considered confusing, and thus require careful interpretation during forensic investigation. Information from this study can prove useful in interpreting witness descriptions of vehicle topcoat colors for more reliable statements.
